# Arnica/Levisticum 6x comp. can alleviate musculoskeletal symptoms in breast cancer patients undergoing endocrine therapy: a case series

**DOI:** 10.3389/fonc.2026.1758527

**Published:** 2026-05-08

**Authors:** Silvia T. Erni, Jana Ertl, Héloïse May, Fabian Grossmann, Ursula Wolf

**Affiliations:** Institute of Complementary and Integrative Medicine, University of Bern, Bern, Switzerland

**Keywords:** arnica, breast cancer, case series, endocrine therapy, levisticum, musculoskeletal symptoms, symptom management

## Abstract

Breast cancer is the most common malignancy among women worldwide, and endocrine therapy (ET) is crucial for reducing recurrence and mortality in hormone receptor–positive cases. However, ET is often associated with adverse effects, especially musculoskeletal symptoms (MSS) such as arthralgia and myalgia, which impair daily functioning, decrease quality of life, and lead to early therapy discontinuation. Although some pharmacological and non-pharmacological options are available for managing ET-induced MSS, their effectiveness is limited, and so far, no general recommendations exist. In the context of integrative oncology, which combines conventional and complementary approaches, anthroposophic medicine has been proposed as a supportive strategy to address these challenges. This case series investigated the potential of the anthroposophic medicinal product *Arnica/Levisticum 6x comp.* for alleviating ET-induced MSS. Three patients with hormone receptor-positive breast cancer, all undergoing ET and experiencing significant MSS unresponsive to conventional management, were included. Clinical data were collected over at least three years of follow-up. The severity of musculoskeletal pain was evaluated using patient reporting and a visual analogue scale (VAS). All three patients reported marked and rapid improvement in ET-associated MSS after beginning treatment with *Arnica/Levisticum 6x comp*. Symptom relief was typically achieved within weeks, with an average reduction of 2–4 points on the VAS. Notably, symptom recurrence was observed during periods of medication interruption, and symptoms consistently resolved upon reintroduction of *Arnica/Levisticum 6x comp.*, suggesting a beneficial therapeutic effect. Throughout the observation period, the medication was well tolerated, with no adverse effects reported. Patients experienced improvements in daily functioning and overall quality of life, which facilitated continued adherence to ET. This case series provides preliminary evidence for the effectiveness and safety of *Arnica/Levisticum 6x comp.* as a therapeutic option for managing ET-induced MSS in breast cancer patients. The sustained symptom relief, favorable tolerability, and long-term safety suggest that this anthroposophic medication may serve as a useful adjunct in supportive care, improving quality of life and promoting continued adherence to ET.

## 1 Introduction

Breast cancer is the most common malignancy among women worldwide (1). For estrogen receptor (ER) positive and progesterone receptor (PR) positive breast cancer adjuvant endocrine therapy (ET) is recommended (2, 3). Endocrine therapy for 5 years significantly lowers breast cancer recurrence by about 40% and mortality by about 30% (4). Unfortunately, despite its benefits, only about 50% of women with breast cancer adhere to ET for the recommended time (5–7). One of the most prominent side effects of ET is musculoskeletal symptoms (MSS) that occur in approximately 13.5% of patients receiving tamoxifen and over 40% of those treated with aromatase inhibitors (8, 9). Naturally, patients are less likely to follow through with treatment when faced with severe side effects (10, 11). About 82.7% of postmenopausal women who discontinued ET with letrozole did so due to adverse side effects, in particular because of MSS (12, 13).

Various medications have been explored for mitigating MSS induced by aromatase inhibitors, including vitamin D (14, 15), glucosamine and chondroitin (16) and a low dose of oral prednisolone (17), but the current level of evidence is low. Non-pharmacological methods (including exercise, acupuncture, yoga) show promising results, but so far, no established recommendations exist.

This case series investigated the use of the anthroposophic medicinal product *Arnica/Levisticum 6x comp.* (ampoules, Weleda) (*Ar/Lev 6x comp.*) for the management of ET-induced MSS. Anthroposophic medicine is practiced by licensed physicians worldwide and is part of integrative medicine, which combines conventional and complementary approaches, addressing both physical and psychosocial factors (18).

## 2 Case description

Three pre- or postmenopausal women with hormone receptor-positive breast cancer (ER+/PR+), all undergoing ET and experiencing significant MSS refractory to conventional management (regular physical activity) were referred to our institute for consultation on integrative oncology. The anthroposophic medicinal product *Ar/Lev 6x comp.* was administered, aiming to alleviate MSS and thereby improve ET tolerance. Case selection was based on methodological criteria. Long-term follow-up and documented repeated discontinuation phases were required to assess the temporal relationship between treatment and symptoms.

The severity of MSS was evaluated using patient reporting and a visual analogue scale (VAS), with scores ranging from 0 (no pain) to 10 (maximal pain). Additionally, regular oncology evaluations took place according to the follow-up schedule. Clinical data were collected by the physician in regular patient consultations over a minimum follow-up period of three years.

This case series report was written following the CARE guidelines (19). Ethical review and approval were not required for this case series in accordance with local legislation and institutional requirements. All patients provided written informed consent for participation and publication of the case series.

### 2.1 Case patient A

A 64-year-old (1959) postmenopausal Caucasian woman was referred to our institute with a diagnosis of invasive ductal breast carcinoma and high-grade ductal carcinoma *in situ* left pT1c pN1a (1/13) L0 V0 Pn0 G3 R0 with ER+ (90%), PR+(10%), Her2 negative (first diagnosed in May 2019). Secondary diagnoses were meniscal tear due to degenerative damage (2022), post-cholecystectomy by cholecystolithiasis (2017).

The patient was a healthy-weight woman (BMI 23.8) with no known allergies or prior medications. She was a non-smoker, did not consume alcohol, had 3 children and was divorced. She reported no known family history of cancer (health status of maternal and paternal grandmothers unknown). She did not engage in any specific sports but walked about 8 km daily und worked regularly in her garden. She used to work as a nurse in an 80% part-time position in 2019 (retired in January 2023).

Following the breast cancer diagnosis, she underwent curative treatments (**Supplementary Table 1**) and started ET with letrozole 2.5 mg/day in March 2020. Three months into therapy, she developed morning joint stiffness and start-up pain, especially in the knees and hands limiting her quality of life and ability to play the guitar, which had been an important personal resource for her. Subcutaneous injections of one ampoule of *Ar/Lev 6x comp.* three times weekly were initiated in June 2020. Regular injections from July 2020 onwards led to a marked reduction of MSS symptoms. In April 2021, the patient reported a very good overall condition without any specific joint complaints, allowing the injection frequency to be reduced to twice weekly.

Voluntary discontinuation of *Ar/Lev 6x comp.* in spring 2023 led to the recurrence of the joint complaints (VAS 4-6/10) which resolved upon resuming the twice weekly regimen (VAS 1-2/10). The twice-weekly dose of *Ar/Lev 6x comp.* proved optimal for this patient, as further reduction (once weekly) negatively impacted her ability to play the guitar for more than one hour.

Being able to play the guitar remains crucial for this patient’s well-being. Treatment with *Ar/Lev 6x comp.* enabled her to pursue this by addressing the MSS, especially in her fingers and wrist joints. No side effects were reported during more than three years of *Ar/Lev 6x comp.* treatment. **Supplementary Table 2** summarizes additional symptoms reported by Patient A and the corresponding prescribed complementary treatments.

### 2.2 Case patient B

A premenopausal 44-year-old (1979) Caucasian woman was referred to our institute with the following diagnoses: Inflammatory Breast Carcinoma left, NST ypT2 ypN1a (1/44) L1 V0 Pn0 G2 R0 with ER+ (90%), PR+ (1%), Her2 zero, Ki-67 5% (first diagnosed in August 2019). Secondary diagnoses were migraine from about 9–15 years old and COVID-19 disease in 2021. No known musculoskeletal diagnoses.

The patient had a healthy weight (BMI 23.5, remained stable), a potential allergy to co-amoxicillin antibiotics, did not smoke, drank occasionally one glass of alcohol but had stopped 4 months prior to neoadjuvant chemotherapy, had 2 children, was married, no cancer history in the family, was physically active at least once or twice a week (Pilates and other sports). She was working in the gastronomy sector at an 20% part-time position.

In the context of her principal diagnosis, she underwent curative treatment (**Supplementary Table 3**). Endocrine therapy with leuprorelin 3.75 mg/month was started in June 2020. Around 2–3 weeks later, the patient reported morning stiffness and start-up pain in her articulations (up to max VAS 9/10). Subcutaneous *Ar/Lev 6x comp.* three times weekly was initiated, leading to improvement of pain (VAS 4/10) by the end of August 2020. Because the aromatase inhibitor anastrozole 1 mg/d was added to the treatment in August 2020, additional *Arnica/Levisticum 3x comp.* (dilution, Weleda) (*Ar/Lev 3x comp.*) drops were introduced as a preventive measure (1–3 times 20 drops per day on days without injections, i.e. 4x weekly). A voluntary three-week discontinuation of the *Arnica/Levisticum* treatment between August and December 2020 caused severe MSS relapse with almost immobility due to pain. It resolved within one week after restarting *Arnica/Levisticum* administrations. In December 2020 the patient reported very good symptom control with *Ar/Lev 6x comp.* subcutaneou*s* injections three times weekly and additional *Ar/Lev 3x comp.* 3x20 drops per day at least 4 times weekly.

In May 2022, the patient reported that she had discontinued the *Ar/Lev 6x comp.* injections in the first quarter of 2022 but continued taking the *Ar/Lev 3x comp.* drops. The MSS remained bearable. The patient subsequently also stopped *Ar/Lev 3x comp.* (time frame unknown), which led to a relapse of ET-induced MSS (VAS 4/10). Once she resumed taking the *Ar/Lev 3x comp.* drops (1-3x 20 drops per day when needed) the symptoms improved within a few days (VAS 2/10), as reported in December 2022. To simplify the therapy, only the subcutaneous injections with *Ar/Lev 6x comp.* were continued. They were considered more practical since they require less frequent administration compared to the drops.

The patient reported less symptoms in summer and mild exacerbations in winter. Therefore, she independently adjusted the injection frequency to once weekly in summer and twice to three times weekly in winter, achieving stable symptom control without further need for *Ar/Lev 3x comp.* drops. In December 2024, the patient reported very good management of MSS (VAS ≤4/10) with *Ar/Lev 6x comp.* In case of periods of prolonged discontinuation, pain would increase to VAS 6-8/10.

Since August 2020, the external oncologist’s reports have regularly noted only mild joint pain, and no significant adverse effects and overall good tolerability of ET. In December 2023, it was specifically stated that owing to the supportive therapy the ET -induced symptoms are well tolerated by the patient.

The patient considered the treatment as an important tool for controlling her MSS and reported not having experienced any side effects from *Arnica/Levisticum* treatment. **Supplementary Table 4** summarizes additional symptoms reported by Patient B and the corresponding prescribed complementary treatments.

### 2.3 Case patient C

A postmenopausal 56-year-old (1967) Caucasian woman was referred to our institute with the following diagnoses: multifocal invasive breast carcinoma NST left (first diagnosed in February 2020) pT2 pN0 CMx L0 V0 Pn0 G3 R0 Stadium IIA ER+ (99%), PR+ (20%), Ki-67 5-30%, HER2 negative. Secondary diagnoses were post-varicose operation in both legs (2002 and 2008), vestibular neuritis (2010) and tinnitus in both sides of unclear etiology (since 2006). No known musculoskeletal diagnoses.

The patient had a healthy weight (BMI 21.9); with no known allergies, did not smoke, drank 1–2 glass of alcohol per week, was divorced since 10 years, had 3 children, lived with a partner, was physically active (sport sessions twice a week for at least one hour, additional walking/hiking for 2–3 hours once a week), one paternal and one maternal aunt had a history of breast cancer; no cancer known among siblings or parents, worked as a hospital sterilization assistant at a 80% part-time position.

Following her principal diagnosis, she underwent curative treatment (**Supplementary Table 5**). Endocrine therapy with letrozole 2.5 mg was started in early October 2020. By the third week, the patient experienced severe insomnia linked to an increase in hot flushes. At approximately 5–6 weeks, she also developed arthralgia and morning stiffness. Subcutaneous injections of *Ar/Lev 6x comp.* three times weekly were initiated, leading to marked improvement within three weeks. Due to good symptom control *Ar/Lev 6x comp.* could be reduced to twice weekly in March 2022. Over almost two years symptoms remained minimal. In February 2023, discontinuation of *Ar/Lev 6x comp.* increased MSS (VAS 3-4/10), which improved quickly (VAS 1-2/10) after resuming the injections twice weekly in June 2023. In November 2023, the symptoms were still well controlled.

External oncological evaluation in July 2023 confirmed good overall tolerance to ET. The patient has accommodated the under therapy minor ET-associated adverse effects (joint pain) which do not restrict her daily functioning. The patient considered the *Arnica/Levisticum* therapy as beneficial. The stabilizing and relieving effects were clearly noticeable, particularly through their absence during discontinuation phases. No adverse reactions to *Ar/Lev 6x comp.* medication were reported. **Supplementary Table 6** summarizes additional symptoms reported by Patient C and the corresponding prescribed complementary treatments.

The case characteristics for all three patients are presented in **Table 1**. A timeline is provided in the supplementary material (**Supplementary Figure 1**).

**Table 1:** Case characteristics.

Item	Patient A	Patient B	Patient C
Physical activity	Walks 8 km/day, gardening	Sports 1–2×/week (Pilates or Aquafit), Walks 30 min/day	Sports 3x/week (sport sessions and walking/hiking)
Endocrine therapy (dose, initiation date)	Letrozole (2.5 mg/d, 03/2020)	Leuprorelin (3.75 mg/month, 06/2020), anastrozole (1 mg/d, 08/2020)	Letrozole (2.5 mg/d, 10/2020)
Reported onset of MSS	After 3 months of ET	After 2–3 weeks of ET	After 5–6 weeks of ET
MSS severity without *Ar/Lev 6x comp.*	Morning stiffness and pain mainly in knees and hands	Morning stiffness and severe pain in articulations	Morning stiffness and arthralgia, mainly wrists, feet and elbows
	2023: VAS 4-6/10	06/2020: VAS 9/10 2022: VAS 4/10 2024: VAS 6-8/10	2023: VAS 3-4/10
*Ar/Lev 6x comp.*: initial dose frequency	3x/week, 1 amp. Break of 6 days after 14 amp.	3x/week, 1 amp. Break of 6 days after 14 amp.	3x/week, 1 amp. Break of 6 days after 14 amp.
Effect of *Ar/Lev 6x comp./Ar/Lev 3x comp.* treatment	Pain improvement (from VAS 4-6/10 to 1–2/10)	Pain improvement (End of 2020: from very severe (no VAS) to VAS 4/10 2022: from VAS 4/10 to 2/10 2024: from VAS 6-8/10 to <4/10)	Pain improvement (2023: from VAS 3-4/10 to1-2/10)
*Ar/Lev 6x comp.*: modified dose frequency	2x/week, 1 amp. (after 10 months)	temporary *Ar/Lev 3x comp.* drops (after 2 months) 2x/week, 1 amp. (after 18 months) Seasonal adaptation (after three years): winter 2-3/week, 1 amp., summer 1x/week, 1 amp.	2x/week, 1 amp. (after 24 months)
Adverse reactions	None	None	None
Patient perspective on *Ar/Lev 6x comp.*	November 2023: Very good symptom reduction and enabled playing instruments; high benefit	April 2021/June 2024: Beneficial for symptom control and very important for ET-adherence	November 2022: Clear benefit for well-being and ability to perform unrestricted recreational sport

## 3 Discussion

Sustaining ET for at least five years is vital for optimal outcomes in breast cancer patients (20). Unfortunately, discontinuation rates are between 15-60%, depending on the type of ET, and significant side effects play an important role in the decision to interrupt or discontinue ET (21–23). Joint pain induced by ET is frequently cited as the leading cause for premature discontinuation. Approximately 50% of patients will report new onset or worsening joint pain 1 year after therapy initiation (24), approximately 30% of patients discontinue therapy after 1 year, and only 50%–68% of patients remain fully compliant with therapy after 3 years. Unfortunately, this leads to an increased risk of breast cancer relapse and mortality (25, 26).

No established evidence-based treatment exists for treating and preventing ET-induced MSS. The effectiveness of therapies remains uncertain, and study heterogeneity and methodological limitations prevent reliable guidance for clinical practice (27). Among the strategies evaluated, non-pharmacologic approaches, particularly structured exercise and physical therapy, show the most consistent evidence for reducing pain and improving function, although also for this evidence the overall quality is limited (27). To address this unmet need, anthroposophic medicine within integrative oncology represents a promising approach.

To our knowledge, this is the first published case series that documents a positive course over an observational period of at least 3 years. In the presented cases, ET led to the development of MSS in breast cancer patients within weeks, despite the patients being physically active. MSS pain intensities ranged from moderate to severe. Subcutaneous *Ar/Lev 6x comp.*, three times weekly (or less), resulted in rapid symptom relief (about 2–4 points on the VAS), with long-term stabilization. Treatment interruptions in all cases led to symptom relapses, while reinitiation restored symptom control, suggesting a therapeutic effect of *Ar/Lev comp*. A 2-point decrease on a 0–10 pain intensity scale, such as the VAS, is considered a clinically important improvement for individual patients (28). Administration of *Ar/Lev 6x comp.* exceeded this threshold and thus provided a clinically meaningful benefit, further supported by the patient’s narrative appraisal. The treatment regimen and dosing adjustments were guided by clinical expertise and were individualized according to symptom severity and patient preference. No adverse reactions were reported by the patients. Safety data by the manufacturer confirms exceptionally low incidence of adverse events. The evaluation by our oncological physician colleagues, especially in Patient B (highest MSS), confirmed good tolerability of ET and treatment adherence due to the supportive medication of integrative medicine. The presented case series reflect a highly favorable harm–benefit profile of *Ar/Lev 6x comp*.

*Ar/Lev 6x comp*. is an anthroposophic medicinal product containing potentized plant and animal-derived substances. One milliliter contains 333 mg *Apis mellifica (Ph.eur.Hom.) 6x*, 333 mg *Arnica montana ex planta tota Rh (HAB) 6x*, 333 mg *Levisticum Rh (HAB) 6x, 9 mg Natrii chloridum and Aqua ad injectabile.* It is intended for subcutaneous injection. *Ar/Lev 6x comp*. solution for injection is produced in compliance with the Anthroposophic Pharmaceutical Codex APC (29), aligning with Good Manufacturing Practice (GMP) standards. It is registered as a complementary medication in Germany, Austria, and Switzerland, with its first authorization in Switzerland granted in 1969.

The individual components of *Ar/Lev 6x comp*. exert anti-inflammatory and analgesic effects: *Arnica montana* has anti-inflammatory (30, 31) and anti-edematous properties (32), *Apis mellifica* (bee venom) has dose-dependent anti-inflammatory and anti-nociceptive effects (33, 34) and *Levisticum* exhibits anti-inflammatory activity (35).

No specific dietary modifications or complementary movement therapies, such as yoga, Tai Chi or Qi Gong were adopted during the observation period. At the same time, all three patients remained physically active throughout endocrine treatment (**Table 1**). Analgesics (paracetamol or NSAIDs) were used short-term for conservative management of a meniscal condition (Patient A) and for headaches (Patients B and C), but not for MSS. Complementary medications (other than *Ar/Lev 6x comp.)* were either used temporarily (based on symptom presentation), or continuously (including during discontinuation phases) in the context of the underlying cancer diagnosis (e.g., *Viscum album* therapy) (**Supplementary Tables 2**, **4**, **6**). However, individual patient courses showed that apart from *Ar/Lev 6x comp*., none of the other medications influenced MSS, as the symptom burden remained unchanged whether these medications were administered or not.

### 3.1 Limitations

Case reports are based on clinical records that are collected during regular consultations. While infrequent follow-up visits can limit patients’ detailed recall of medications use and their effects on symptoms over prior weeks or months, the consistency of the findings in this case series still allow for a meaningful overall interpretation.

Symptom severity was assessed descriptively using the patients’ reporting and the VAS. It has been previously shown that the VAS, when used repeatedly, is a reliable indicator of the subjectively perceived pain burden at present and in recent months (36).

Endocrine therapy-related side effects vary with treatment duration. Arthralgia often develops within the first 6 weeks of ET and may worsen over the first year (37, 38). Longer follow-up assessments show that mild joint pain may increase over 12 months and then decline, while moderate pain can steadily increase up to 18 months (39), suggesting symptoms rarely resolve spontaneously. In our case series, ET-related symptom burden over three years closely paralleled the initiation and discontinuation of *Ar/Lev 6x comp*, rather than of the usual fluctuation pattern typically associated with ET treatment.

Although placebo effects cannot be ruled out in this case series, several observations speak against it as the sole explanation for the clinical improvements: Symptoms did not resolve immediately after *Ar/Lev 6x comp.* treatment initiation but improved gradually and remained stable over several years, which is atypical for placebo responses in chronic musculoskeletal pain (40).

Results from a case series are not to be generalized, but we would like to emphasize that these three cases reflect many years of clinical experience with this treatment, and it is plausible that other patients suffering from ET-induced MSS may similarly benefit from *Ar/Lev 6x comp.* therapy. We report sustained improvement over a follow-up period of at least three years. Although we did not test this, there is no reason to believe that the therapeutic effect would diminish before completion of the full course of ET (minimum of five years). Nonetheless, to confirm the effects of *Ar/Lev 6x comp.* on ET-induced MSS in a larger population, the compound ideally is to be tested in randomized controlled trials with standardized repeated pain assessments and evaluation of ET adherence over a minimum of five years.

## 4 Conclusion

This case series provides encouraging preliminary evidence that the anthroposophic medicinal product *Ar/Lev 6x comp*. may safely and effectively reduce ET-induced MSS in breast cancer patients. All three patients experienced durable symptom relief without discontinuing ET over the observational period, indicating excellent tolerability and long-term safety. By alleviating pain, improving quality of life, and supporting adherence to ET, *Ar/Lev 6x comp*. may serve as a valuable complementary approach in integrative oncology, offering a favorable harm–benefit profile and potentially contributing to better long-term outcomes. This is the first case series to document sustained benefits for at least three years.

Take-home messages:

In these three breast cancer patients *Ar/Lev 6x comp.* treatment:

1. alleviated ET-induced pain and stiffness in the musculoskeletal system.
2. caused no side effects.
3. improved functioning and quality of life.
4. was suited for long-term use and supported ET-adherence.

## Data Availability

The data analyzed in this study is subject to the following licenses/restrictions: The dataset is confidential. It is derived from the physician’s consultation notes, which are protected by the patient-physician relationship, and therefore cannot be made publicly available. All data presented in the manuscript have been fully anonymized. Requests to access these datasets should be directed to JE, jana.ertl@unibe.ch.
