# Supplementary material for: Arnica/Levisticum 6x comp. can alleviate musculoskeletal symptoms in breast cancer patients undergoing endocrine therapy: a case series
Source: Front Oncol. 2026 May 8;16:1758527. doi: 10.3389/fonc.2026.1758527 (PMC13193916; doi:10.3389/fonc.2026.1758527)
Supplement: Supplementary file 1 [file DataSheet1.pdf]

frontiers

Supplementary Material

All patients were treated with an Integrative Oncology approach, combining conventional oncological therapies with complementary treatments

Supplementary Table 1. Patient A: Oncological treatment

**Time**	**Intervention/Treatment**
May 2019	Initial diagnosis
June 2019	Tumorectomy and axillary nodes resection level I and II
July - September 2019	Four cycles of adjuvant chemotherapy (doxorubicin and cyclophosphamide)
October - December 2019	Twelve administrations of adjuvant chemotherapy (paclitaxel)
February - March 2020	Adjuvant radiotherapy on the entire breast (in total 50 Gy)
March 2020	High-dose-rate brachytherapy boost (in total 15 Gy)
Since March 2020	Endocrine therapy (ET) (letrozole 2.5 mg, planned until 2024)

Supplementary Table 2. Patient A: Complementary treatments

Date	Products	Posology
July - December 2019	*Iscador P c Hg Serie 0* (mistletoe)	3x/week, s.c. after 14 ampoules skip one injection day
December 2019 - March 2021	*Iscador P c Hg Serie I* (mistletoe)	3x/week, s.c. after 14 ampoules break of 6 days
Since April 2021	*Iscador P c Hg Serie I* (mistletoe)	2x/week, s.c. after 7 ampoules break of 6 days
Since July 2019	*Gentiana cp* (globules, Wala, AMP)	3-5x10 globules additionally in case of nausea, for digestion, on demand
September 2019 - January 2020	*Stibium met praep 6x* (ampoule (amp.), Weleda, AMP)	2x/week s.c. against chemotherapy-induced peripheral neuropathy, preventive measure and therapeutic
December 2019	*Bryophyllum Argento cultum rh 3x* (dilution (dil.), Weleda, AMP)	Single prescription, 1-3x15 drops for insomnia, on demand
February - March 2020	*Argentum/Urtica comp* (gel, Wala, AMP)	Topical application in case of radiotherapy-induced erythema
June - August 2020	*Solutio silicea cp 3x* (dil., Weleda, AMP)	25 drops for skin and nail regeneration for 2-3 months
June 2020 - April 2021	*Rheum rhaponticum 0,6% or 0,4%* (ointment, Weleda, AMP)	2-4x/week against genital dryness
June 2022	Phytotherapeutic drop mixture	single prescription, 3x20 drops for digestion
Since June/July 2022	*Cyanara scolymus* (tincture, Ceres)	1-3x 5 drops, for digestion of fats, on demand (external prescription)

Caption: ampoule (amp.), subcutaneous (s.c.), tablet (tbl), dilution (dil.), anthroposophic medicinal product (AMP)

Supplementary Table 3. Patient B: Oncological treatment

Time	Intervention/Treatment
August 2019	Initial diagnosis
August 2019 - January 2020	Four cycles of neoadjuvant chemotherapy (cyclophosphamide, epirubicin and 5-fluoruracil)
February - May 2020	Twelves administrations of neoadjuvant chemotherapy (paclitaxel)
May 2020	Mastectomy left and resection of the axillary nodes level I and II
July - August 2020	Adjuvant radiotherapy with thorax, supra- and infraclavicular region, axillary and internal breast nodes region left and bradytherapy boost (in total 66.4 Gy)
Since June 2020	ET (leuprorelin 3,75 mg/month, premenopausal state)
Since August 2020	ET (anastrozole 1 mg/day, planned for 10 years in total)
2022	Breast reconstruction

Supplementary Table 4. Patient B: Complementary treatment

Dates	Products	Posology
Since 2019	*Cimifemin uno* (medicinal black cohosh 6,5 mg)	1 tbl/day, against ET-induced hot flushes
December 2019 - August 2020	*Iscador P c Hg Serie 0* (mistletoe)	3x/week, s.c. after 14 ampoules skip one injection day
August 2020 - February 2022	*Iscador P c Hg Serie 0* (mistletoe)	3x/week, s.c. after 14 ampoules break of 6-13 days
Since May 2022	*Iscador P c Hg Serie 0* (mistletoe)	2x/week, s.c., after 7 ampoules break of 6 days
December 2019 - May 2020	*Gentiana cp* (globules, Wala, AMP)	3-6x 10 globules for appetite, on demand
February - June 2020	*Stibium D6* (amp., Weleda, AMP)	2-3x/week s.c. preventive measure and therapeutic in chemotherapy-induced peripheral neuropathy
March 2020 - December 2023	*Sepia comp* (dil., Weleda, AMP)	3x 10-15 against ET-induced mood swings, on demand
June 2020 - December 2023	*Rheum rhaponticum 0,6%* (ointment, Weleda, AMP)	2-3x/week topical genital application (against genital dryness)
Since June 2020	*Rheum rhaponitcum 0,4%* (vaginal supp., Weleda, AMP)	2-3x/week topical genital and vaginal application (against ET-induced genital and vaginal dryness)
June - August 2020	*Argentum/Urtica cp* (gel, Wala, AMP)	Topical application in case of radiotherapy-induced erythema
December 2021 - January 2022	*Quarz D60* (amp., Weleda, AMP)	Single prescription, 1x/week for better convalescence
December 2022	*Euphorium comp NS* (homeopathic remedy)	Single prescription, nasal spray temporarily for rhinitis

Caption: ampoule (amp.), subcutaneous (s.c.), tablet (tbl), dilution (dil.), anthroposophic medicinal product (AMP), endocrine therapy (ET)

Supplementary Table 5. Patient C: Oncological treatment

Time	Intervention/Treatment
February 2020	Initial diagnosis
May 2020	Left breast central segment resection with pectoralis fascia, cranio-caudal resection, left axilla sentinel node excision
May 2020	Post-resection latero-caudal and caudal left breast
June - August 2020	Four cycles of adjuvant chemotherapy (doxorubicin and cyclophosphamide)
September 2020	Brachytherapy boost of the left breast (in total 15 Gy)
September - November 2020	Radiotherapy of the left breast (in total 50 Gy)
Since October 2020	ET (letrozole 2,5mg/day, planned for 5 years minimum)

Supplementary Table 6. Patient C: Complementary treatments

Dates	Products	Posology
Started before July 2020	*Cimifemin uno* (medicinal black cohosh 6,5mg)	1 tbl/day (against ET-induced hot flushes)
July - October 2020	*Iscador P c Hg Serie 0* (mistletoe)	3x/week, s.c. after 14 ampoules skip one injection day
November 2020 - February 2022	*Iscador P c Hg Serie 0* (mistletoe)	3x/week, s.c. after 14 ampoules break of 6 days
Since March 2022	*Iscador P c Hg Serie 0* (mistletoe)	2x/week, s.c. after 7 ampoules break of 6 days
July - September 2020	*Antimonit/Rosae aeth. Cp* (gel, Wala, AMP)	Topical application several times a day in case of oral mucositis, aphthosis
July - August 2020	*Gentiana cp* (globules, Wala, AMP)	3-6x 10 globules for slow digestion and bloating, on demand
February - November 2020	*Argentum/Urtica* (gel, Wala, AMP)	Local application in case of radiotherapy-induced erythema
September 2020 - March 2023	*Sepia cp* (dil. Weleda, AMP)	2-3x 15 drops for ET-associated hot flushes and mood swings, on demand
October - December 2020	*Valeriana comp* (dil., Ceres, mother tincture)	3x 5 drops at night on demand in case of insomnia
November 2020 - March 2022	*Cimicifuga cp* (dil., Weleda, AMP)	3-4x 20 drops against ET-associated hot flushes, on demand
November 2020	*Comfrey* (ointment, Dr Andres, physiotherapeutic remedy)	Single prescription: topical application in case of arthralgia after sports

Caption: ampoule (amp.), subcutaneous (s.c.), tablet (tbl), dilution (dil.), anthroposophic medicinal product (AMP), endocrine therapy (ET)

Supplementary Figure 1. Timeline

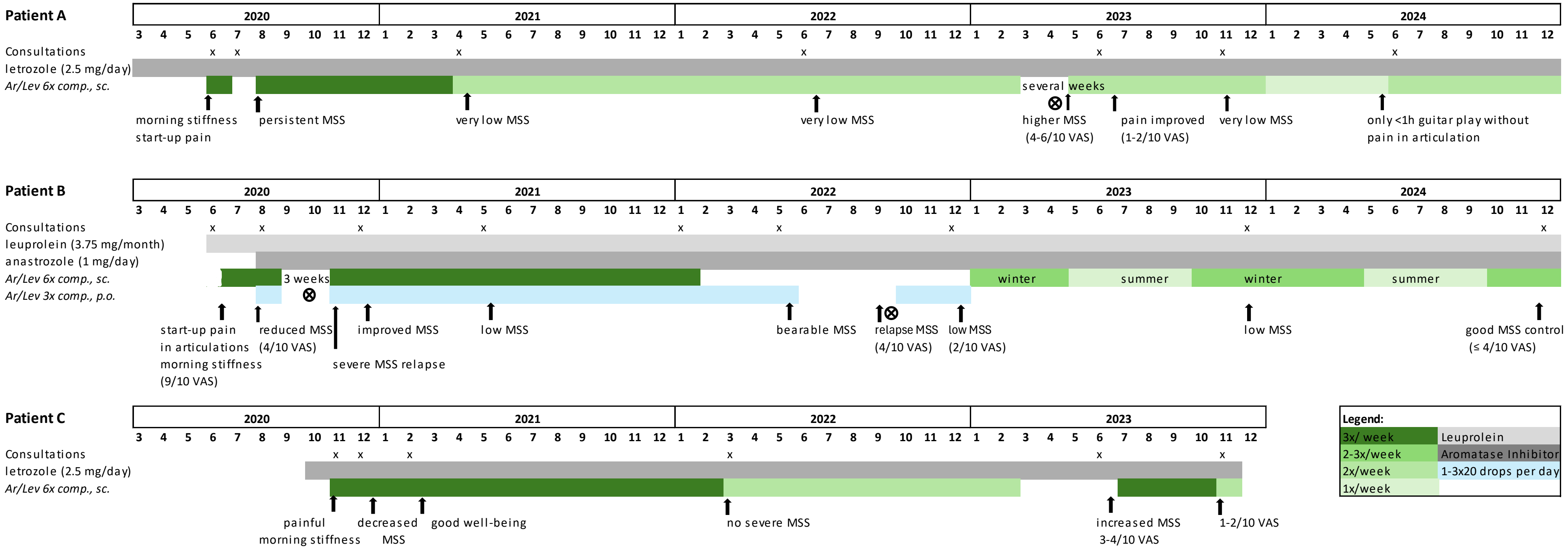

Caption: Timeline (year and months) of ET and *Ar/Lev 6x comp*., s.c. and *Ar/Lev 3x comp*. p.o. administrations with description of MSS severity (s.c.: subcutaneous, p.o.: per os).

⊗: Exact dates of discontinuation periods were not reported in detail by the patient
